# Diffuse intracranial calcification, deep medullary vein engorgement, and symmetric white matter involvement in a patient with systemic lupus erythematosus

**DOI:** 10.1111/cns.13250

**Published:** 2019-10-25

**Authors:** Yin‐Xi Zhang, Hong‐Fu Li, Yang Zheng, Lei Wu, Zhi‐Ying Wu, Mei‐Ping Ding

**Affiliations:** ^1^ Department of Neurology Second Affiliated Hospital School of Medicine Zhejiang University Hangzhou China

Systemic lupus erythematosus (SLE) is a chronic relapsing‐remitting autoimmune disease with multiple autoantibodies causing damage to different target organs. Central nervous system is commonly involved, with a myriad of clinical and radiologic features always complicating the diagnosis. In this study, we report an atypical case of SLE with brain involvement showing a variety of unique and novel neuroimaging findings.

A 53‐year‐old woman presented with slurred speech, deep voice, and gait instability for over 1 year. She had a 13‐year history of SLE and had been treated with corticosteroid. Neurological examination revealed a female with the moon face, dysarthria, bradykinesia, rigidity, hyperreflexia of the upper extremities, and abnormal tandem gait.

Routine laboratory examinations including parathyroid hormone and serum calcium were normal except for immunologic tests, which showed antinuclear antibodies titer 1:80 (++), antidouble‐stranded deoxyribonucleic acid antibodies 563.8 IU/mL (reference range < 100 IU/mL), antiribonucleic protein antibodies (++), anti‐Sjögren's‐syndrome‐related antigen A antibodies (+++), and anti‐Ro52 antibodies (+++). Cerebrospinal fluid assay revealed no abnormalities. Computed tomography (CT) scan of the chest, ultrasounds of the lymph nodes, and abdomen were normal. Brain magnetic resonance imaging revealed patchy lesions in centrum semiovale and periventricular regions, with prolonged signals on T1 and T2‐weighted images and normal signals on diffusion‐weighted images, suggesting leukoencephalopathy; additional lesions bilaterally in the basal ganglia, thalamus, and cerebellar dentate nucleus showed shortened signals on T1 and T2 images, as well as low signals on susceptibility weighted imaging (SWI), with medullary veins slightly dilated (Figure 1). Brain magnetic resonance angiography was normal. Head CT demonstrated multiple patchy and punctate lesions in the bilateral lenticular nucleus and cerebellar dentate nucleus, as well as radiating lesions along the periventricular area (Figure 2). Those lesions have a symmetric distribution with a CT unit of around 300 HU, which were consistent with calcification. Targeted next‐generation sequencing was unrevealing, ruling out hereditary leukoencephalopathy, and intracranial calcification‐related hereditary diseases.

**Figure 1:**
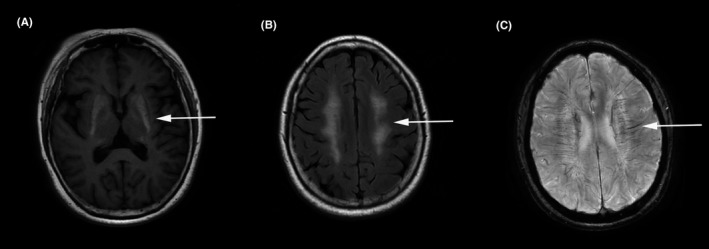
Brain magnetic resonance imaging showed lesions in the bilateral basal ganglia with short T1 signals (A, arrow), lesions across the bilateral centrum semiovale with long T2 signals (B, arrow), as well as slight dilations of the deep medullary vein along the bilateral periventricles on susceptibility weighted imaging (C, arrow)

**Figure 2:**
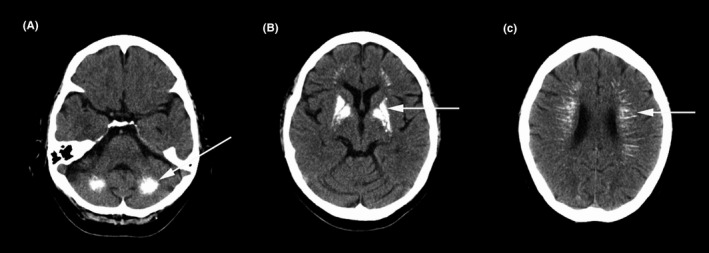
Head computed tomography scan showed symmetric calcified lesions in the dentate nucleus (A, arrow) and lenticular nucleus (B, arrow) bilaterally, as well as radiating high‐density lesions along the periventricular region (C, arrow)

A final diagnosis of central nervous system lupus (CNSL) was made. After consulting with the rheumatologist, the dosage of methylprednisolone was increased from 8 to 16 mg/d. In follow‐up, related antibodies turned negative 10 days after initialization of treatment. The patient felt that the symptoms had improved and reported no disease progression 1.5 years later.

Neurological manifestations are seen in around 50% of SLE patients.^1^, ^2^ Symptoms vary and always include vascular headache, seizures, stroke, abnormal movements, cognitive impairment, and psychiatric abnormalities. There is a great heterogeneity in neuroradiological findings as well. In the acute phase, manifestations suggesting infarction, hemorrhage, and inflammatory lesions could be seen, while leukoencephalopathy and atrophy are also present in the chronic phase.^2^ White matter abnormalities in SLE patients often manifest as asymmetric, multifocal small lesions, with fronto‐parieto‐occipital subcortical lesions most commonly seen.^3^ The leukoencephalopathy in this case, interestingly, appeared extensive and symmetric, which was uncommon.^4^ Intracranial calcifications were rarely reported in SLE and were believed to be unrelated to the severity of neuropsychiatric symptoms.^5^ Among all the reported calcified brain regions, basal ganglia were most commonly affected, with few cases reporting calcifications in the white matter, cerebellum, and even the cortex.^6^, ^7^ Our patient with multiple calcifications with such a diffuse pattern is indeed a rarity, which could explain her main extrapyramidal‐cerebellar symptoms and signs. Differential diagnosis included endocrine causes of abnormal calcium metabolism (hypoparathyroidism or pseudohypoparathyroidism), infectious diseases, neoplastic diseases, and other hereditary neurological diseases. These conditions may show similar manifestations especially for imaging results, but there was no evidence to support such diagnoses in this case. Another novel finding in our case lies in the dilation of deep medullary veins. This finding was evident on SWI and was further corroborated by the pattern of radial periventricular calcifications on CT scan. To our knowledge, the periventricular radial calcification along deep medullary veins has not previously been reported, supported that calcium deposits in perivascular spaces and small vein walls.^7^ It is now hypothesized that brain calcifications in CNSL are partly due to vessel abnormalities, though the exact pathogenesis is still unknown.^6^, ^8^, ^9^, ^10^ Considering the unique imaging features of this case, we speculate that the expansion of the medullary veins, gradual formation of intracranial calcification, and white matter injury were due to the localized ischemia in SLE caused by immune‐related microvascular damage during the prolonged period of disease.

Although CNSL is not uncommon, it is rare to see a patient with a combination of atypical neuroradiological findings (diffuse intracranial calcification, deep medullary vein engorgement, and symmetric white matter involvement). The results reported here broaden the known clinical and radiologic features. Our patient exhibited a good response to corticosteroids after dose adjustment and remains stable to date, further highlighting the importance of recognizing the potentially treatable disorder.

## CONFLICT OF INTEREST

The authors declare no conflict of interest.
